# The Protective Effect of Brazilian Propolis against Glycation Stress in Mouse Skeletal Muscle

**DOI:** 10.3390/foods8100439

**Published:** 2019-09-25

**Authors:** Tatsuro Egawa, Yoshitaka Ohno, Shingo Yokoyama, Takumi Yokokawa, Satoshi Tsuda, Katsumasa Goto, Tatsuya Hayashi

**Affiliations:** 1 Laboratory of Sports and Exercise Medicine, Graduate School of Human and Environmental Studies, Kyoto University, Kyoto 606-8501, Japan; takumi.yokokawa@gmail.com (T.Y.); tsuda.satoshi.55u@st.kyoto-u.ac.jp (S.T.); tatsuya@kuhp.kyoto-u.ac.jp (T.H.); 2 Laboratory of Health and Exercise Sciences, Graduate School of Human and Environmental Studies, Kyoto University, Kyoto 606-8501, Japan; 3 Laboratory of Physiology, School of Health Sciences, Toyohashi SOZO University, Toyohashi 440-8511, Japan; yohno@sozo.ac.jp (Y.O.); s-yokoyama@sozo.ac.jp (S.Y.); gotok@sepia.ocn.ne.jp (K.G.); 4 Department of Physiology, Graduate School of Health Sciences, Toyohashi SOZO University, Toyohashi 440-8511, Japan

**Keywords:** advanced glycation end products, anti-glycation, glycative stress, glyoxalase, methylglyoxal, cytokine

## Abstract

We investigated the protective effect of Brazilian propolis, a natural resinous substance produced by honeybees, against glycation stress in mouse skeletal muscles. Mice were divided into four groups: (1) Normal diet + drinking water, (2) Brazilian propolis (0.1%)-containing diet + drinking water, (3) normal diet + methylglyoxal (MGO) (0.1%)-containing drinking water, and (4) Brazilian propolis (0.1%)-containing diet + MGO (0.1%)-containing drinking water. MGO treatment for 20 weeks reduced the weight of the extensor digitorum longus (EDL) muscle and tended to be in the soleus muscle. Ingestion of Brazilian propolis showed no effect on this change in EDL muscles but tended to increase the weight of the soleus muscles regardless of MGO treatment. In EDL muscles, Brazilian propolis ingestion suppressed the accumulation of MGO-derived advanced glycation end products (AGEs) in MGO-treated mice. The activity of glyoxalase 1 was not affected by MGO, but was enhanced by Brazilian propolis in EDL muscles. MGO treatment increased mRNA expression of inflammation-related molecules, interleukin (IL)-1β, IL-6, and toll-like receptor 4 (TLR4). Brazilian propolis ingestion suppressed these increases. MGO and/or propolis exerted no effect on the accumulation of AGEs, glyoxalase 1 activity, and inflammatory responses in soleus muscles. These results suggest that Brazilian propolis exerts a protective effect against glycation stress by inhibiting the accumulation of AGEs, promoting MGO detoxification, and reducing proinflammatory responses in the skeletal muscle. However, these anti-glycation effects does not lead to prevent glycation-induced muscle mass reduction.

## 1. Introduction

The skeletal muscle is the largest organ that contributes to maintaining physical locomotive function. It is also a major site of glucose and lipid metabolism and an endocrine organ with myokine secretions [1]. A number of epidemiological studies revealed that people with type 2 diabetes tend to have lower muscle strength and mass [2]. The potential underlying mechanism of this skeletal muscle dysfunction is linked to hyperglycemia, chronic inflammation, and oxidative stress [2].

Glycation is a biochemical process through which reducing sugars like glucose react and bond non-enzymatically with proteins. Glycation stress, which is caused by glycation and includes the formation of advanced glycation end products (AGEs) and a subsequent dysfunction of proteins and/or cellular signaling [3], are considered related with the progress of muscle dysfunctions. It has been reported that elevated AGEs in the blood or skin are negatively correlated with muscle mass, grip strength, and glucose tolerance in the elderly [4,5,6,7,8] and patients with diabetes [9]. Our recent study demonstrated that AGEs suppressed formation of myotubes in C2C12 skeletal muscle cells by deteriorating cellular signal transduction of protein synthesis and suggested that AGEs inhibited skeletal muscle formation and maturation [10]. Furthermore, serum AGE levels are related to diabetic complications in children and young adults with type 1 diabetes [11,12,13], thus indicating that glycation stress might affect skeletal muscle function regardless of age. In fact, our previous study revealed that the consumption of an AGE-rich diet for 16 weeks in young mice led to degenerative changes in skeletal muscle, including low muscle mass, low grip strength, low force relative to muscle mass, and muscle fatigability [14]. Furthermore, AGEs treatment in skeletal muscle has been illustrated to induce insulin resistance in young male and female rodents [15,16]. Therefore, inhibiting glycation stress is considered an effective strategy for preventing skeletal muscle dysfunction regardless of age.

AGEs lead to the activation of different signaling pathways mediated by several cell surface receptors. The activation of receptors for AGEs (RAGE) is considered as a major mediator of AGE pathogenicity [17,18]. Although the recruitment of RAGE stimulates myogenesis that is important for skeletal muscle development, the chronic stimulation of RAGE, due to high concentration of AGEs, causes myopathy through inflammatory responses [19]. In addition to RAGE, toll-like receptor 4 (TLR4) is involved in AGE-mediated inflammatory responses, such as cytokine production [20]. The interaction between AGEs-RAGE leads to activation of intracellular nuclear factor-κ B and subsequently increases the expression of several proinflammatory cytokines, including tumor necrosis factor-α (TNFα) and interleukin (IL)-6 [21]. Furthermore, AGEs stimulates the secretion of IL-6 through RAGE and/or TLR4 in macrophages [22]. A recent study has also demonstrated that AGEs-induced inflammatory responses occur via IL-1β in human placental cells [23]. These proinflammatory cytokines are known factors of muscle wasting [24] and insulin resistance [25], and thus the suppression of AGEs-associated inflammatory responses can be a target of maintaining muscle functions.

Propolis, a natural resinous substance produced by honeybees, is traditionally used in herbal medicine and has recently been suggested to possess several biological properties including anticancer, antioxidant, and anti-inflammatory activities [26]. The wide diversity of plant species used by bees as resin sources for propolis production determines its chemical diversity by region. Among propolis of various production area, Brazilian propolis contains a number of phenolic compounds such as artepillin C, p-coumaric acids, and kaempferide [27,28], and has become a popular health supplement due to its many biological properties [29]. Recent studies have reported that several polyphenol substances exert anti-glycation functions by inhibiting the formation of AGEs, promoting their degradation, and by exerting an antagonizing effect on AGE receptors [30]. This suggests that Brazilian propolis may possess an anti-glycation capacity and contributes to maintaining skeletal muscle functions. Previous studies demonstrated that European poplar type of propolis have anti-glycation activity in vitro [31,32,33]. However, no reports have investigated the anti-glycation effects of Brazilian propolis and its efficacy in vivo.

In the present study, we aimed to examine the protective effect of Brazilian propolis against glycation stress in the skeletal muscle. To this end, we subjected the skeletal muscles of mice to glycation stress using methylglyoxal (MGO), a precursor of AGEs, for 20 weeks and investigated the effect of Brazilian propolis on alleviation of this stress.

## 2. Materials and Methods

### 2.1. Animals and Treatment

Twenty-four male C57BL/6NCr mice (4-weeks-old) were purchased from Shimizu Breeding Laboratories (Kyoto, Japan). The mice were placed in a room maintained at 22–24 °C with a 12:12 h light/dark cycle. After 1 week of adjustment, the mice were randomly divided into four groups (*n* = 6/group): (1) Normal diet (AIN-93G; Oriental Koubo, Tokyo, Japan) + drinking water (N), (2) Brazilian propolis (0.1%)-containing diet + drinking water (PRO), (3) normal diet + MGO (0.1%)-containing drinking water (MGO), and (4) Brazilian propolis (0.1%)-containing diet + MGO (0.1%)-containing drinking water (MGO + PRO). The Brazilian propolis powder of ethanol extracts (LY-009), standardized to contain a minimum of 8.0% artepillin C was obtained from Yamada Bee Company, Inc. (Okayama, Japan). The Brazilian propolis was originated from Baccharis dracunculifolia of Southeast Brazil. The nutritional information of AIN-93G and Brazilian propolis powder of ethanol extracts is listed in Table 1. The doses of propolis and methylglyoxal, and their duration of intake were determined by previous experimental studies [34,35]. For each group, all mice were housed in a single cage and provided free access to food and drinking water for 20 weeks. Body weight was measured once every two weeks. Food and fluid intakes were measured during two consecutive days every two weeks and averaged as grams per day per mouse.

At the end of the study period, the slow-twitch soleus muscle and fast-twitch extensor digitorum longus (EDL) muscles and tibia were collected from each mouse under anesthesia using mixtures of medetomidine hydrochloride (0.3 mg/kg), midazolam (4.0 mg/kg), and butorphanol (5.0 mg/kg). All animal protocols were carried out in accordance with the Guide for the Care and Use of Laboratory Animals by the National Institutes of Health (Bethesda, MD, USA) and were approved by the Kyoto University Graduate School of Human and Environmental Studies (approval number: 28-A-2, approval date: 2016.3.29).

### 2.2. Anti-Glycation Assay

The anti-glycation activity of propolis was performed using the Albumin Glycation Assay Kit (AAS-AGE-K01, Cosmo Bio, Tokyo, Japan). Briefly, propolis was dissolved in dimethyl sulfoxide at a concentration of 0, 0.1, 1, 10, and 100 mg/mL, and the solutions were incubated with 50 mM glyceraldehyde and bovine serum albumin solutions for 48 h at 37 °C. The fluorescence of AGEs was estimated using a fluorescence microplate reader equipped with a 355 nm excitation filter and 460 nm emission filter. Inhibitory effects of AGE formation were expressed as percent change relative to the value of a solution containing 20 mM aminoguanidine.

### 2.3. Measurement of MGO-Derived AGE Content

The MGO-derived AGE content in muscles was measured using an OxiSelect Methylglyoxal Competitive ELISA Kit (STA-811, Cell Biolabs, Milpitas, CA, USA) according to the manufacturer’s protocol.

### 2.4. Measurement of Glyoxalase 1 Activity

The activity of glyoxalase 1 in muscles was measured using a Glyoxalase I Activity Assay Kit (Colorimetric) (K591-100, BioVision, San Diego, CA, USA) according to the manufacturer’s protocol.

### 2.5. Real-Time RT-PCR Analysis

A separate set of muscle samples were subjected to RT-PCR analysis, which was performed as previously described [36]. Total RNA was extracted from frozen muscles using the RNeasy Mini Kit (Qiagen, Venlo, Netherlands). RNA was reverse-transcribed into complementary DNA (cDNA) using PrimeScript RT Master Mix (Perfect Real Time) (Takara Bio, Kusatsu, Japan). Synthesized cDNA was subjected to real-time RT-PCR (Step One Real Time System, Applied Biosystems, Carlsbad, CA, USA) using SYBR Premix Ex Taq II (Takara Bio, Kusatsu, Japan) and then analyzed using StepOne Software v2.3 (Applied Biosystems, Foster City, CA, USA). Relative fold change of expression was calculated by the comparative CT method. β-actin and ribosomal protein S18 (Rps18) was used as an internal standard. Primers used were as follows: Interleukin-1β (IL-1β), 5’-TCCAGGATGAGGACATGAGCAC-3’ (forward) and 5’-GAACGTCACACACCAGCAGGTTA-3’ (reverse); IL-6, 5’-CCACTTCACAAGTCGGAGGCTTA-3’ (forward), and 5’-TGCAAGTGCATCATCGTTGTTC-3’ (reverse); toll-like receptor 4 (TLR4), 5’-TCCTGTGGACAAGGTCAGCAAC-3’ (forward) and 5′-TTACACTCAGACTCGGCACTTAGCA-3’ (reverse); receptor for AGE (RAGE), 5’-AGCCACTGGAATTGTCGATGAG-3’ (forward), and 5’-GCTGTGAGTTCAGAGGCAGGA-3’ (reverse); β-actin, 5’-CATCCGTAAAGACCTCTATGCCAAC-3’ (forward), and 5’-ATGGAGCCACCGATCCACA-3’ (reverse); and Rps18, 5’-TTGGTGAGGTCAATGTCTGCTTT-3’ (forward), and 5’-AAGTTTCAGCACATCCTGCGAGT-3’ (reverse).

### 2.6. Statistics

All values were expressed as means ± SE. For each group of data, normality (the Kolmogorov–Smirnov test) and equal variance tests (Levene’s test) were performed and data that were not normally distributed were log-transformed before the analysis of variance (ANOVA). The statistical significance of differences in body weight, food intake, and fluid intake between groups was determined via a repeated-measures ANOVA. The statistical significance of differences in muscle weight, MGO-derived AGEs content, and mRNA expression was analyzed using two-way ANOVA with propolis and MGO as the main factors. In the event of significant main effects and/or interactions, post hoc Tukey-Kramer tests were performed. Differences between groups were considered statistically significant at *p* < 0.05. All statistical analyses were performed using the Ekuseru-Toukei 2012 software (Social Survey Research Information, Tokyo, Japan).

## 3. Results

### 3.1. Anti-Glycation Effects of Brazilian Propolis In Vitro

**Figure 1:**
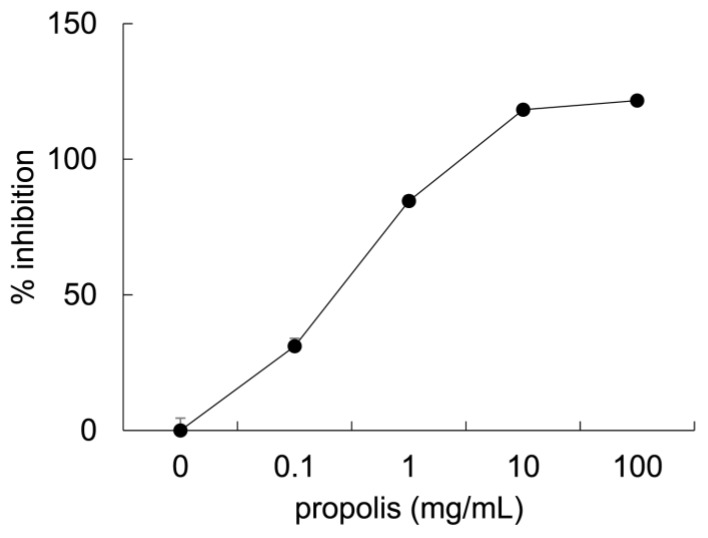
The inhibitory effect of Brazilian propolis used at different concentrations (0, 0.1, 1.0, 10, and 100 mg/mL) on formation of advanced glycation end products (AGEs). Values are means ± SE; *n* = 4/group. Values are expressed as percent change relative to the value of aminoguanidine.

The inhibitory activity of Brazilian propolis against formation of AGEs was evaluated by measurement of fluorescent AGEs formed by glyceraldehyde and bovine serum albumin (Figure 1). Propolis inhibited the formation of fluorescent AGEs (0 mg/mL, 0 ± 4.53%; 0.1 mg/mL, 31.1 ± 2.87%; 1.0 mg/mL, 84.5 ± 2.00%; 10 mg/mL, 118 ± 0.62%; 100 mg/mL, 122 ± 1.79%, means ± SE, *n* = 4/group).

### 3.2. The Effect of Brazilian Propolis on Body Weight, Food and Fluid Intake, and Muscle Weight

Body and muscle weights and food and fluid intake are presented in Table 2 and Figures S1–S3. Repeated measures ANOVA did not reveal significant differences in the body weights among the groups (*p* = 0.070). Food intake was significantly different among the groups (*p* = 0.0004); specifically, MGO + PRO group had lower food intake than all other groups (*p* = 0.007 vs. N; *p* = 0.003 vs. PRO; *p* = 0.001 vs. MGO). Fluid intake was significantly different among the groups (*p* = 0.0004); in that, the PRO (*p* = 0.010), MGO (*p* = 0.043), and MGO + PRO (*p* = 0.0003) groups had lower fluid intakes than N group. Two-way ANOVA revealed that MGO, but not propolis, had a significant main effect on EDL muscle weight normalized to tibia length (propolis, *p* = 0.69; MGO, *p* = 0.039) (Table 2) and muscle cross sectional area (CSA) (propolis, *p* = 0.95; MGO, *p* = 0.042) (Table S1). No significant main effects were observed for soleus muscle weight normalized to tibia length (propolis, *p* = 0.054; MGO, *p* = 0.086) (Table 2) and muscle CSA (propolis, *p* = 0.18; MGO, *p* = 0.13) (Table S1). However, propolis and MGO showed a large (η^2^ = 0.16) and moderate (η^2^ = 0.12) effect size in soleus muscle mass as calculated using η^2^, respectively.

### 3.3. Brazilian Propolis Suppressed the Accumulation of MGO-Derived AGEs in the Skeletal Muscle In Vivo

**Figure 2:**
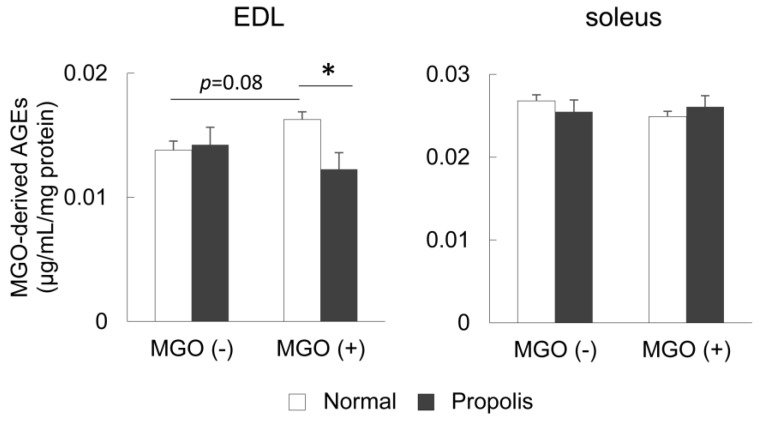
The content of methylglyoxal (MGO)-derived advanced glycation end products (AGEs) in skeletal muscles. The extensor digitorum longus (EDL) and soleus muscles were dissected from mice treated with or without Brazilian propolis (0.1%)-containing diet or MGO (0.1%)-containing drinking water for 20 weeks. Values are means ± SE; *n* = 5–6/group. * *p* < 0.05 between the groups.

The content of MGO-derived AGEs in EDL and soleus muscles was measured to evaluate the effect of Brazilian propolis on accumulation of AGEs in the skeletal muscle in vivo. In the EDL muscle, two-way ANOVA revealed a significant interaction (*p* = 0.020); specifically, the content of MGO-derived AGEs following MGO treatment tended to increase (*p* = 0.08), but it had a large effect size as calculated using Cohen’s d (d = 1.54). Brazilian propolis ingestion suppressed this accumulation (*p* = 0.003) (Figure 2). In the soleus muscle, no significant alterations in the content of MGO-derived AGEs was observed according to ANOVA (MGO, *p* = 0.59; propolis, *p* = 0.97) (Figure 2).

### 3.4. Brazilian Propolis Enhanced Glyoxalase 1 Activity in The Skeletal Muscle

**Figure 3:**
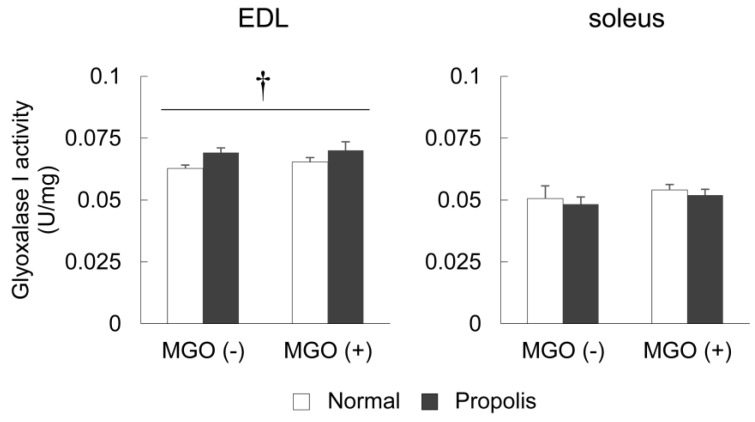
The activity of glyoxalase 1 in skeletal muscles. EDL and soleus muscles were dissected from mice treated with or without Brazilian propolis (0.1%)-containing diet or MGO (0.1%)-containing drinking water for 20 weeks. Values are means ± SE; *n* = 5–6/group. †, significant main effect between diets (normal and propolis).

To evaluate the ability of Brazilian propolis to detoxify MGO in the skeletal muscle, the activity of glyoxalase 1, a dicarbonyl compound eliminating enzyme, was measured in the EDL and soleus muscles. In the EDL, two-way ANOVA revealed a significant main effect of propolis, but not MGO (propolis, *p* = 0.038; MGO, *p* = 0.49) (Figure 3). In the soleus muscle, no significant alterations in glyoxalase 1 activity was observed via ANOVA (MGO, *p* = 0.25; propolis, *p* = 0.47) (Figure 3).

### 3.5. Brazilian Propolis Suppressed MGO-Induced mRNA Expression of Inflammatory-Related Molecules in The Skeletal Muscle

**Figure 4:**
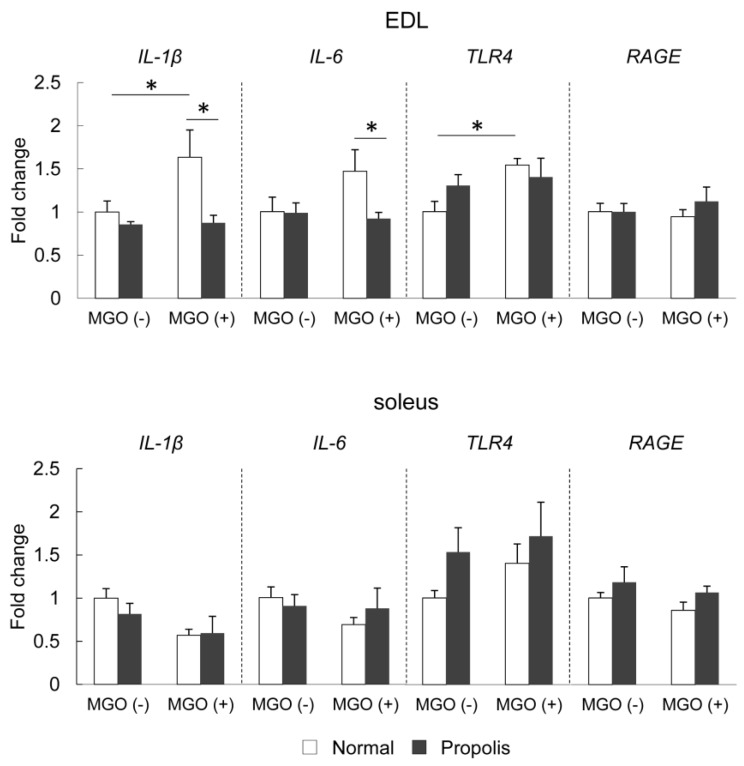
mRNA expression of interleukin (IL)-1β, IL-6, toll-like receptor 4 (TLR4), and receptor for AGEs (RAGE) in skeletal muscles. The EDL and soleus muscles were dissected from mice treated with or without propolis (0.1%)-containing diet or MGO (0.1%)-containing drinking water for 20 weeks. Data of IL-1β in the EDL muscle were log-transformed for normal distribution before analysis of variance (ANOVA). Values are means ± SE; *n* = 3–6/group. * *p* < 0.05 between the groups.

To evaluate the effect of propolis on inflammatory responses, the mRNA expression of proinflammatory cytokines, IL-1β and IL-6, and AGEs-related receptors, TLR4 and RAGE, were measured in the EDL and soleus muscles (Figure 4). In the EDL muscle, two-way ANOVA revealed significant effects on IL-1β, Il-6, and TLR4 expression. MGO treatment significantly increased the mRNA expression of IL-1β (*p* = 0.037); however, the ingestion of Brazilian propolis suppressed this increase (*p* = 0.006). MGO treatment tended to increase the mRNA expression of IL-6, and propolis ingestion suppressed this effect in MGO-treated mice (*p* = 0.036). MGO treatment significantly increased the mRNA expression of TLR4 (*p* = 0.028), but no change was observed under propolis ingestion (*p* = 0.20). The mRNA level of RAGE in the EDL muscle was not altered by either MGO (*p* = 0.28) or propolis (*p* = 0.41). In the soleus muscle, no significant alterations in the mRNA expression of IL-1β (MGO, *p* = 0.078; propolis, *p* = 0.44), IL-6 (MGO, *p* = 0.80; propolis, *p* = 0.34), TLR4 (MGO, *p* = 0.21; propolis, *p* = 0.38), and RAGE (MGO, *p* = 0.33; propolis, *p* = 0.16) were observed via ANOVA (Figure 4).

## 4. Discussion

The current study revealed several novel findings regarding the effect of Brazilian propolis on glycation stress in the skeletal muscle. Firstly, Brazilian propolis inhibited the formation of AGEs in vitro (Figure 1). Secondly, the 20-week ingestion of Brazilian propolis suppressed the accumulation of MGO-derived AGEs (Figure 2), promoted activity of glyoxalase 1 (Figure 3), and attenuated mRNA expressions of proinflammatory cytokines IL-1β and IL-6 (Figure 4) in the EDL but not the soleus muscle.

Glycation stress is suppressed by several mechanisms such as inhibition of AGEs formation, MGO formation, and oxidative stress, detoxification of MGO, and blocked activation of AGEs receptors [30]. To date, many researchers have evaluated the inhibitory effect of natural compounds on the formation of AGEs, and many natural plants are confirmed to reduce glycation stress by inhibiting this formation [37,38]. In this study, we provided evidence for the inhibitory capacity of Brazilian propolis on formation of AGEs in vitro (Figure 1). To the best of our knowledge, this is the first study to demonstrate Brazilian propolis-induced anti-glycation activity. In accordance with this finding, European propolis, which differ from Brazilian propolis in terms of raw materials and components, have been revealed to inhibit glucose-derived and _D_-ribose-derived AGEs production [31,32,33]. These findings suggest that various types of propolis have the capacity to inhibit AGEs formation in vitro.

We also provided a subsequent confirmation for the inhibitory effect of Brazilian propolis on formation of AGEs in vivo by showing that Brazilian propolis led to suppression of MGO-derived AGE accumulation in the skeletal muscle of MGO-loaded mice (Figure 2). This protective effect was seen in the fast-type EDL muscle but not the slow-type soleus muscle. Our previous study demonstrated that a 16-week glycation stress induced by a high-AGE diet in mice promoted the accumulation of AGEs in the EDL but not the soleus muscle [14]. Furthermore, another research has shown that the accumulation of AGEs in the diabetic rat skeletal muscle was greater in fast-type muscle [39]. These findings suggest that fast-type muscles are susceptible to AGEs and that Brazilian propolis improves the inhibitory capacity against AGE formation in fast-type muscle. The potential mechanisms regarding the greater susceptibility of fast-type muscles to AGEs have been described. First, slow-type muscles have a higher protein turnover rate than fast-type muscles [40,41], thus indicating that AGEs are more easily broken down in slow-type muscle than fast-type muscle, and fast-type muscles have a tendency to accumulate AGEs. Second, fast-type muscles are more susceptible to changes in nutrients and hormones than slow-type muscles [42], thus indicating that fast-type muscles are more sensitive to AGEs and propolis than slow-type muscles. However, considering the finding that MGO tended to affect muscle mass with a large effect size in soleus muscle (Table 2), additional examinations using other muscles are needed to clear the fiber-type specific susceptibility to glycation stress.

Brazilian propolis increased muscle mass of soleus almost significantly (*p* = 0.054) with a moderate effect size (η^2^ = 0.12), raising a possibility that Brazilian propolis has a hypertrophic effect in soleus muscle regardless of glycation stress. However, there was no significant difference in the calculated muscle CSA (Table S1), indicating that propolis-induced increase in soleus muscle mass was not caused by hypertrophy. In this regard, Brazilian propolis might stimulate glycogen accumulation, and thereby led to muscle mass gain, because it has been shown that Brazilian propolis stimulated glucose uptake in mouse skeletal muscle [43]. However, a previous study has shown that six-week intake of water extract of Korean propolis did not affect glycogen content in the gastrocnemius muscle of rat [44]. Another possibility is that Brazilian propolis increased connective tissue in muscle because it has been shown that propolis stimulated migration and proliferation of fibroblast cells [45]. At present, however, we have no clear explanation for the mechanism by which Brazilian propolis causes gain of soleus muscle mass without hypertrophy.

Detoxification of MGO is also important for reducing glycation stress. MGO is a highly reactive dicarbonyl compound and the major precursor in the formation of AGEs [46,47]. When MGO production exceeds the detoxification capacity, it can modify arginine residues to form MGO-derived AGEs [47]. The most important MGO detoxification system is the glyoxalase system and glyoxalase 1 functions as a rate-limiting enzyme in this system. Under normal physiological conditions, >99% of MGO is metabolized via the glyoxalase system [48]. In the present study, propolis enhanced glyoxalase 1 activity in the EDL muscle (Figure 3), indicating its capability to detoxify MGO, and thereby in inhibition of MGO-derived AGE production. Therefore, in addition to the inhibitory effect of AGE formation, an enhancement of the glyoxalase system mediated by Brazilian propolis may contribute to the inhibitory effect of accumulation of MGO-derived AGEs in the skeletal muscle.

Inflammation is a crucial contributor toward pathology of diseases implicated in skeletal muscle dysfunction [25,49,50]. Binding of AGEs to AGE receptors including RAGE and TLR4 are potent inducers of inflammatory responses [22]. Inhibition of RAGE and TLR4 effectively reversed the AGE-induced inflammatory signaling [22,51]. In the present study, Brazilian propolis showed no effect of mRNA expression of RAGE, but prevented MGO-treated induction of IL-1β, IL-6, and TLR4 (Figure 4). Consistent with this observation, previous studies have shown that propolis inhibits production of IL-1β in human immune cells [52] and IL-6 in murine macrophages [53]. The current study is the first study that shows that Brazilian propolis has a protective effect on AGE-induced inflammatory responses in the skeletal muscle.

Among the various components of Brazilian propolis [27,28], kaempferide [54], ferulic acid [55], and caffeic acid derivatives [56] are established inhibitors of AGE formation. Furthermore, it has been shown that propolis-induced anti-inflammatory responses may occur due to the synergistic effect of its compounds, artepillin C [57], coumaric acid and cinnamic acid [53], and hesperidin, quercetin, and caffeic acid derivatives [52]. Flavonoid compounds also have a stimulating effect on the glyoxalase system and thereby contribute to neuroprotection [58]. Collectively, the protective activity of propolis against glycation stress in the skeletal muscle may be attributed to the combined biological activity of these phenolic compounds.

Food and fluid intakes were significantly affected by propolis and/or MGO treatment (Table 2). Food intake was reduced in the MGO + PRO group compared with that in the other groups, thus suggesting that MGO + PRO group received a lower contribution from propolis. However, the beneficial effects of propolis, including reduced AGEs accumulation and inflammatory responses, were confirmed in this group. Fluid intake was affected by treatment with MGO and/or propolis, but there was no difference between the MGO and MGO + PRO groups, thus indicating that the beneficial effects of propolis in the MGO + PRO group, including reduced MGO-derived AGEs content and inflammatory responses, were not caused by decreased MGO consumption. Therefore, we believe that the difference of food and fluid intakes does not influence the conclusions of this study.

## 5. Conclusions

The present study revealed that Brazilian propolis protects against MGO-induced glycation stress in mouse skeletal muscles. Brazilian propolis inhibits the accumulation of AGEs, promotes MGO detoxification, and reduces the levels of proinflammatory cytokines. However, Brazilian propolis does not prevent glycation-induced muscle mass reduction. These bioactivities of Brazilian propolis may be effective to protect skeletal muscle dysfunctions induced by aging and pathogenesis.

## Figures and Tables

**Table 1:** Nutritional information of AIN-93G and Brazilian propolis powder of ethanol extracts.

Components	AIN-93G (per 100 g)	Brazilian Propolis Powder (per 100 g)
Carbohydrate	63.0 g	4.2 g
Protein	20.0 g	0.7 g
Fat	7.0 g	47.0 g
Mineral	3.5 g	0.4 g
Vitamin	1.0 g	
Calories	400 kcal	758 kcal

**Table 2:** Body weight, food intake, fluid intake, and muscle weight.

	Normal	Propolis	MGO	MGO + Propolis	ANOVA
Initial body weight (g)	17.7 ± 0.8	17.7 ± 0.5	17.6 ± 0.5	17.7 ± 0.4	―
Final body weight (g)	41.1 ± 0.7	41.3 ± 0.8	38.5 ± 0.8	40.4 ± 0.5	*p* = 0.070
Food intake (g/day/mouse)	3.8 ± 0.6 ^†^	3.7 ± 0.4 ^†^	3.7 ± 0.4 ^†^	3.4 ± 0.4	*p* = 0.0004
Fluid intake (g/day/mouse)	3.2 ± 0.4	2.8 ± 0.3 *	2.9 ± 0.3 *	2.7 ± 0.3 *	*p* = 0.0004
EDL weight/tibia (mg/mm)	0.65 ± 0.03	0.67 ± 0.02	0.62 ± 0.02	0.62 ± 0.01	Propolis (*p* = 0.69) MGO (*p* = 0.039)
Soleus weight/tibia (mg/mm)	0.55 ± 0.01	0.59 ± 0.01	0.53 ± 0.02	0.56 ± 0.02	Propolis (*p* = 0.054) MGO (*p* = 0.086)

EDL, extensor digitorum longus; MGO, methylglyoxal; *n* = 4–6/group; * and † indicates *p* < 0.05 vs. Normal and MGO + propolis group, respectively.

## Supplementary Materials

The following are available online at https://www.mdpi.com/2304-8158/8/10/439/s1, Figure S1: Changes in body weight, Figure S2: Changes in food intake, Figure S1: Changes in fluid intake, Table S1: Muscle cross sectional area.
