# Supplementary material for: The Protective Effect of Brazilian Propolis against Glycation Stress in Mouse Skeletal Muscle
Source: Foods. 2019 Sep 25;8(10):439. doi: 10.3390/foods8100439 (PMC6836237; doi:10.3390/foods8100439)
Supplement: Supplementary file 1 [file foods-08-00439-s001.pdf]

Supplementary File

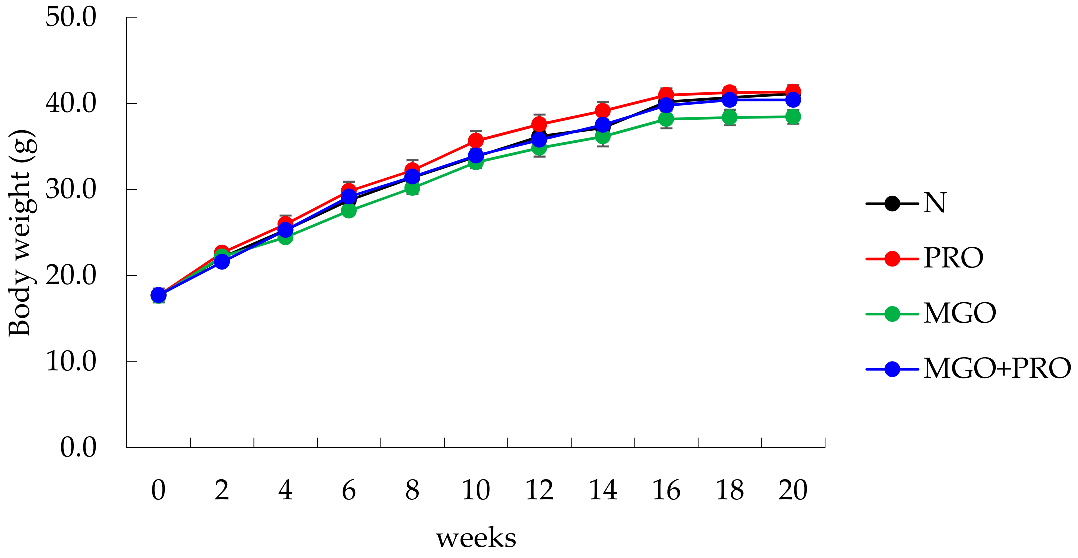

Figure 1. Changes in body weight. Mice were divided into four groups: (1) normal diet + drinking water (N), (2) Brazilian propolis (0.1%)-containing diet + drinking water (PRO), (3) normal diet + MGO (0.1%)-containing drinking water (MGO), and (4) Brazilian propolis (0.1%)-containing diet + MGO (0.1%)-containing drinking water (MGO + PRO). Body weight was measured once every two weeks. Values are means ± SE; n = 5–6/group.

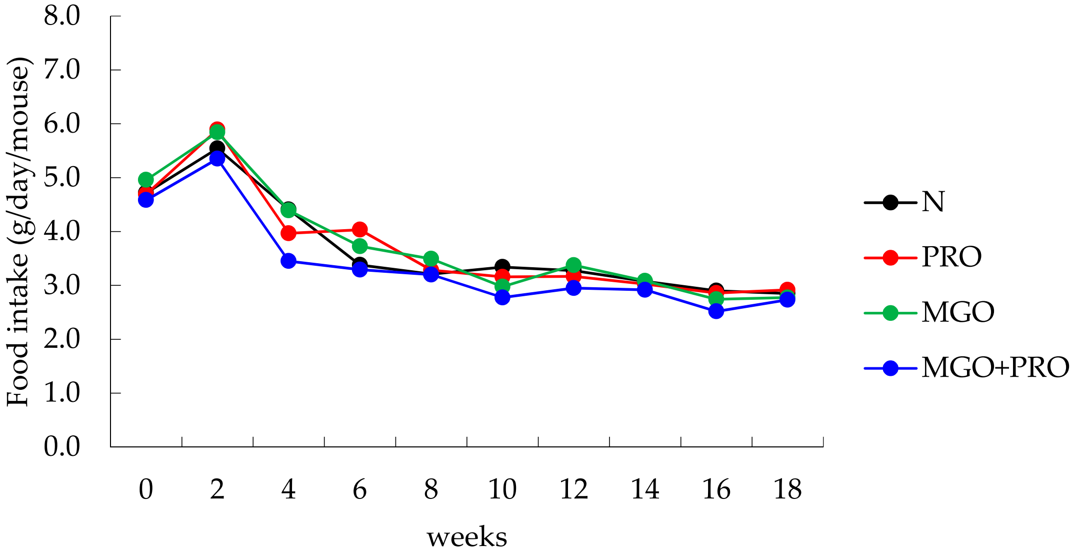

Figure S2. Changes in food intake. For each group, all mice were housed in single cage and provided free access to feeding for 20 weeks. Food intake was measured during two consecutive days every two weeks, and averaged as grams per day per mouse. n = 5–6/group.

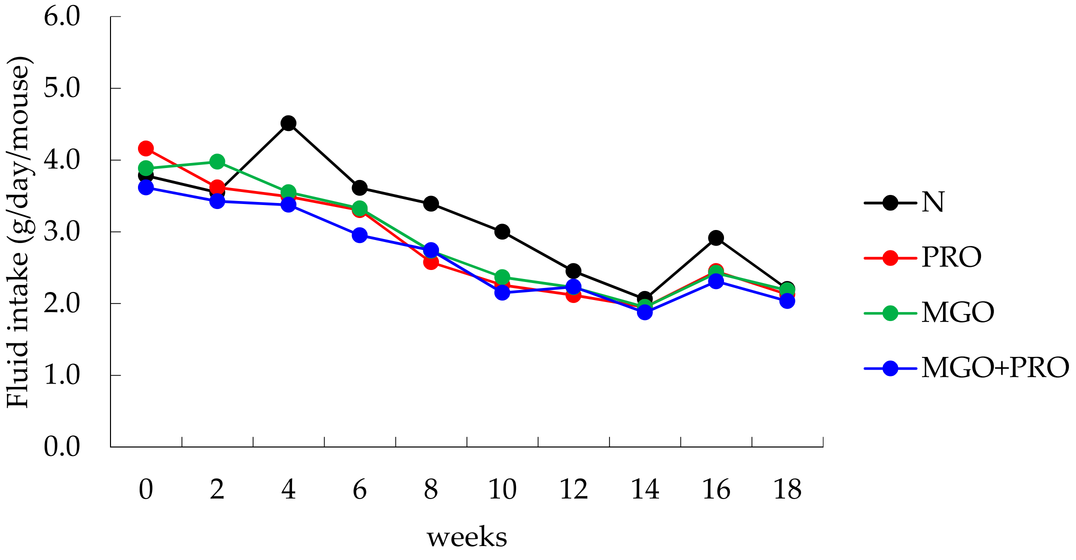

Figure S3. Changes in fluid intake. For each group, all mice were housed in single cage and provided free access to drinking water for 20 weeks. Fluid intake was measured during two consecutive days every two weeks, and averaged as grams per day per mouse. n = 5–6/group.

Table S1. Muscle cross sectional area

	Normal	Propolis	MGO	MGO ＋propolis	ANOVA
EDL (mm^2)	0.86 ± 0.04	0.87 ± 0.02	0.82 ± 0.02	0.80 ± 0.02	Propolis ($p = 0.95$) MGO ($p = 0.042$)
soleus (mm^2)	0.85 ± 0.03	0.91 ± 0.02	0.82 ± 0.04	0.85 ± 0.03	Propolis ($p = 0.18$) MGO ($p = 0.13$)

Muscle cross sectional area was calculated using the standard formula: Cross sectional area (mm^2) = muscle mass (mg)/1.06 (mg/mm^3)/fiber length (mm). 1.06 mg/ mm^3 is the muscle density [1]. The fiber length was calculated as 0.75 × 0.93 × tibia length (mm) in EDL and 0.85 × 0.70 × tibia length (mm) in soleus. 0.75 and 0.85 represent the ratio of the fiber length to the muscle length [2]. 0.93 and 0.70 represent the ratio of the muscle length to tibia length [3].

References

1. Mendez, J., Density and composition of mammalian muscle. *Metabolism* **1960**, *9*, 184-188.
2. James, R.S.; Altringham, J.D.; Goldspink, D.F., The mechanical properties of fast and slow skeletal muscles of the mouse in relation to their locomotory function. *J Exp Biol* **1995**, *198*, 491-502.
3. Clark, K.I.; White, T.P., Morphology of stable muscle grafts of rats: Effects of gender and muscle type. *Muscle Nerve* **1985**, *8*, 99-104.
